# Clinical value of measurement of pulmonary radioaerosol mucociliary clearance in the work up of primary ciliary dyskinesia

**DOI:** 10.1186/s13550-015-0118-y

**Published:** 2015-07-16

**Authors:** Mathias Munkholm, Kim Gjerum Nielsen, Jann Mortensen

**Affiliations:** Department of Clinical Physiology, Nuclear Medicine & PET, Rigshospitalet, Copenhagen University Hospital, DK-2100 Copenhagen Ø, Denmark; Danish PCD Centre, Pediatric Pulmonary Service, Department of Pediatrics and Adolescent Medicine, Rigshospitalet, Copenhagen University Hospital, Copenhagen, Denmark; Department of Medicine, Nuclear Medicine section, The Faroese National Hospital, Torshavn, Faroe Islands

**Keywords:** Mucociliary clearance, Diagnostic test, Primary ciliary dyskinesia

## Abstract

**Background:**

We aimed to evaluate and define the general clinical applicability and impact of pulmonary radioaerosol mucociliary clearance (PRMC) on the work up of patients suspected of having primary ciliary dyskinesia (PCD). In addition, we wanted to evaluate the accuracy of the reference values used in the PRMC test.

**Methods:**

Measurement of PRMC after inhalation of ^99m^Tc-albumin colloid aerosol was carried out on 239 patients (4–75 years of age) during a 9-year period. All were referred to the nuclear medicine department because of clinical suspicion of PCD. The results were compared primarily to results from nasal ciliary function testing, to electron microscopic (EM) examination of the ultrastructure of the cilia, and to the final clinical diagnosis.

**Results:**

Of the 239 patients, 27 ended up with a final clinical diagnosis of definitive PCD. No patients with a PRMC test that was normal or otherwise not consistent with PCD ended up with PCD as final clinical diagnosis (though a minority of patients in this group ended up unresolved in regard to PCD). Forty percent of patients with an abnormal PRMC test ended up with PCD as final clinical diagnosis. Furthermore, the PRMC test had a high rate of conclusive results (90 %). Children <14 years of age with normal PRMC measurements showed significantly faster lung clearance than adults with similarly normal PRMC measurements.

**Conclusions:**

To this date, PRMC is the only test providing evaluation of the mucociliary clearance of the entire lung. Its greatest strength is its ability to reject a suspected PCD diagnosis with great certainty. In our material, this accounted for 2/3 of referred patients. In addition, the test has a high rate of conclusive results. According to our analyses, reference equations on children would benefit from updated data.

## Background

Mucociliary clearance is known to be an important innate defence mechanism against inhaled microbes and irritants. Potentially harmful substances get trapped in the mucus layer lining the airways, and subsequently, the synchronised movement of the cilia propels the mucus to the pharynx where it will be swallowed. This mechanism can be hampered either by conditions affecting the constitution of the mucus (such as cystic fibrosis, chronic obstructive pulmonary disease, or asthma) or by conditions directly affecting the movement of the cilia. This last group of conditions has traditionally been divided into primary and secondary ciliary dyskinesia (PCD and SCD). PCD is a genetic disorder caused by mutations in genes in relation to the axoneme. Clinically, the patients present with cough, dyspnea, and recurrent airway infections starting from early childhood. However, it is both genetically and phenotypically a heterogeneous condition presumably reflecting the molecular complexity of the axoneme, and for this same reason, diagnosing these patients can be challenging. SCD on the other hand includes a variety of temporary, acquired defects of ciliary movement caused by viral or bacterial infection or by certain air pollutants. In some instances, it can be difficult to differentiate SCD from PCD because of overlapping findings in the tests meant for diagnosing PCD [1].

Although there is no definitive evidence that early diagnosis of PCD is beneficial, there is emphasis on finding and treating these patients as early as possible, since early and aggressive treatment of lung infections is thought to be crucial in the prevention of future lung damage [2–4].

The diagnosis relies on the clinical assessment of the patients combined with an array of different tests. These typically include measurement of nasal nitric oxide (nasal NO) where a low NO-value points towards PCD and vice versa, electron microscopic (EM) examination of the ultrastructure of the cilia, and microscopy of ciliary beat pattern and frequency. Genetic testing is developing rapidly, but to this date, only some of the disease-causing genes are known and currently around 65 % of patients could be detected this way [5–7]. Highly specialised modalities used in only few centres are (1) culture of respiratory cells at an air-liquid interface with reanalysis of the ultrastructure of cilia and microscopy of ciliary beat pattern and frequency, (2) immunofluorescence microscopy of respiratory cells, which allows visualisation and localisation or demonstration of the absence of specific proteins in relation to the cilia, and (3) EM tomography, which due to its high resolution may be able to identify ultrastructural defects where normal EM has not determined the abnormality [7–10].

While EM of the cilia and ciliary function testing are both time consuming and somewhat invasive, nasal NO measurement is a fast, non-invasive tool that can generally be used from 5 years of age (modified techniques have been shown to be applicable from 2.5 years) [2, 11] when it comes to targeted case finding and excluding of PCD. But still, there is a need for further investigation when nasal NO values are either inconclusive or point towards PCD [2]. At our centre, measurement of pulmonary radioaerosol mucociliary clearance (PRMC) has been used as part of diagnostic routine tools in the work up of patients suspected of having PCD for over a decade. This method draws on the fact that patients with PCD have impaired mucociliary clearance as a result of abnormal ciliary motility [12]. This abnormal clearance pattern can be visualised for the entire lung using a gamma camera to follow the movement of an inhaled radioaerosol. PRMC is non-invasive, renders a minimal amount of radiation (less than 1 mSv while the yearly background radiation in Denmark is 3–4 mSv), is applicable to children as young as 5 years of age (younger children will normally exhibit a lack of cooperation), and most importantly a normal PRMC can help excluding the diagnosis in patients with symptoms compatible with PCD who might exhibit SCD in the nasal biopsy [13, 14]. In addition, the PRMC test is relatively easy to perform. A simplified protocol for the test is presented in our review published in 2013 [1]. A large number of patients referred for PRMC as work up of PCD diagnosis are children. However, our current reference equations are generated on a group of healthy people >17 years of age. It is still unknown if extrapolating the equations to this younger age group will bias interpretation of results.

The aim of this study is to evaluate and define the general clinical applicability and impact of PRMC on the work up of patients suspected of having PCD. Moreover, the reference values used today will be scrutinised—especially their accuracy when used in the work up of children.

## Methods

### Study design

The study was carried out as a register-based follow-up study. The patients were included continuously over 9 years, and their final clinical diagnosis was evaluated at the end of the study period.

### Patients

In total, 239 patients were included in the study, and all were referred to the nuclear medicine department for PRMC test because of clinical suspicion of PCD. The vast majority of these had PRMC done as part of their work up for PCD in the National PCD Centre in Copenhagen at Rigshospitalet, whereas 64 patients were referred directly from departments of pulmonary medicine in other hospitals, and they were only referred to the National PCD Centre in Copenhagen if the clinical suspicion of PCD sustained after completion of the PRMC test.

Preceding the PRMC test, patients were informed that they had to be without symptoms of acute airway infections 4–6 weeks prior to the test. Some patients had their PRMC test repeated during the study period; but in this paper, when nothing else is stated, only results from the first PRMC test are used.

### PRMC test

The PRMC technique has been described in detail earlier [15]. In brief, the patients inhaled an ultrasonically nebulised ^99m^Tc-albumin colloid with a mass median aerodynamic particle diameter of 3.4 μm. They did 20 tidal breathings with slow inspiration followed without breath hold by forced expiration, and the radioaerosol was administered during the whole inspiration phase. Inhalation volume was not measured. Immediately after the inhalation procedure, the patients rinsed their mouth by gargling three times. Thereafter, lung radioactivity was detected by placing the patients in a supine position against a posteriorly positioned gamma camera, and it was ensured that a target rate of 1000 counts per second was obtained. If not, extra inspirations were made.

Measurement of lung radioactivity was performed for the next 2 h with static acquisitions at 0, 60, and 120 min together with two 20-min dynamic acquisitions (films) in the first hour. This was supplemented with a 15-min static acquisition after 24 h.

Regional ventilation distribution was assessed by an ^81m^Kr-gas scintigram. The initial ^99m^Tc aerosol distribution was compared to this ^81m^Kr ventilation distribution by calculating a penetration index (PI), which reflects how far the radioaerosol had penetrated into the lungs, thereby indicating how far the radioaerosol had to move before being cleared from the airways. Using our reference equations which are based on a cross-sectional study of 53 healthy never-smoking adults (18 to 84 years) [16], the PI, sex, and age of the subject was used to calculate his or her predicted lung retention at 1 and 2 h (pLR_1_ and pLR_2_). These predicted values were then compared to the actual measured lung retention at 1 and 2 h (LR_1_ and LR_2_) corrected for background and physical decay.

The difference between predicted and actual lung retention divided by the standard deviation was calculated as a *Z*-score for LR_1_ and LR_2_, respectively. The upper limit for a normal test was defined as a *Z*-score of >1.645 (i.e., 95 percentile), since an abnormal mucociliary clearance can only be too slow, resulting in too high retention.

**Fig. 1:**
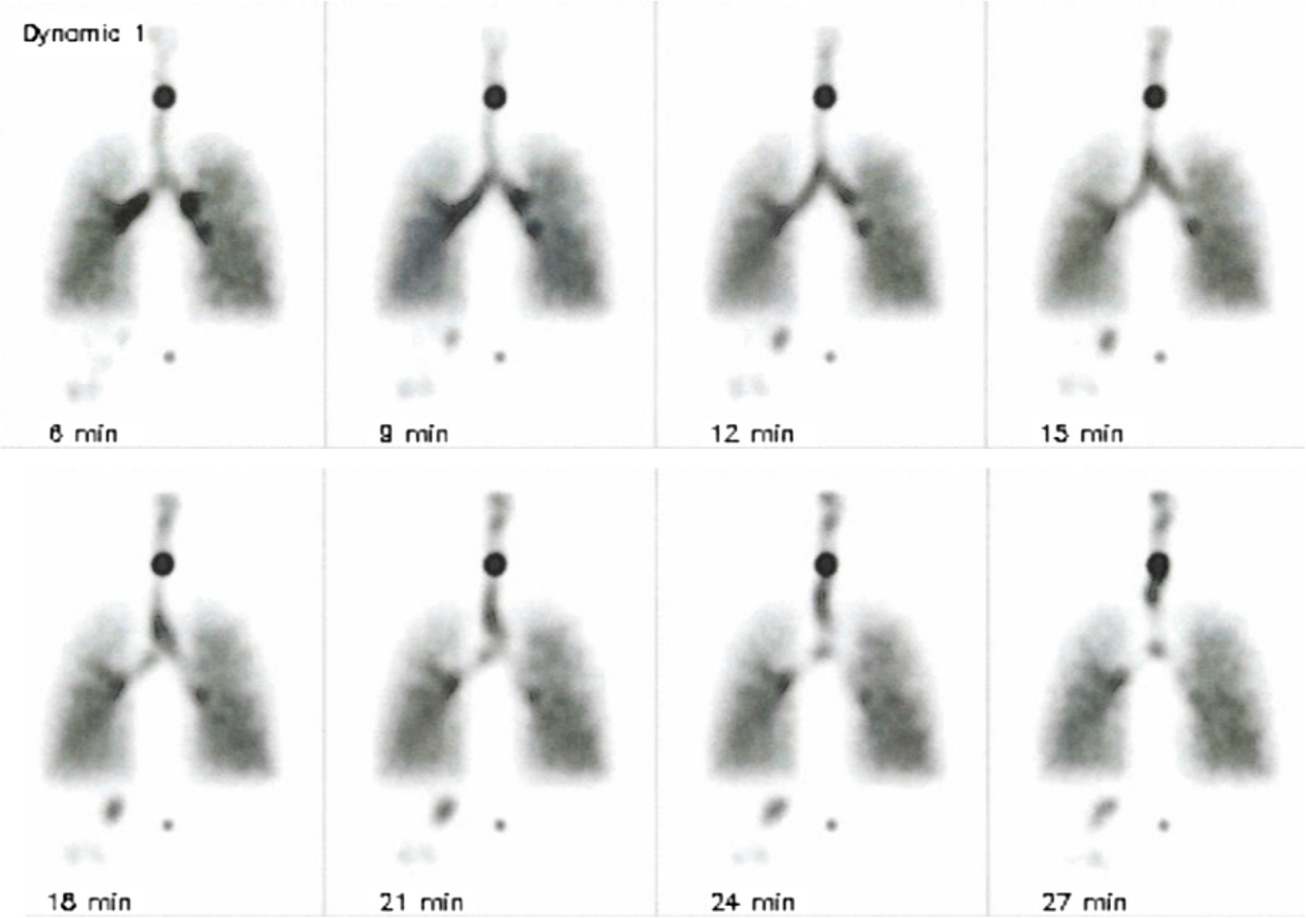
Twenty-minute dynamic posterior acquisition presented as eight pictures each representing 2½ min of normal tracheobronchial bolus transport. Boluses of mucus are seen to ascend from the main stem bronchi to the trachea (*top four pictures*) and further up the trachea (*bottom four pictures*). In the midline, two ^57^Co markers placed above the vertebra C7 and L1 are seen over the trachea and under the lungs, respectively

The two 20-min dynamic acquisitions were used to assess mucus transport in the larger airways. When bolus transport occurred, distinctive radioactive boluses could be seen to move through the main stem bronchi and the trachea (Fig. 1). The movement was visually assessed as normal, abnormal (i.e., slow, absent, or retrograde), or inconclusive.

The interpretation of the PRMC test was based on the following three questions: (1) Were LR_1_ and LR_2_ outside the predicted values? (2) Was the bolus transport in the trachea abnormal? And (3) was focal retention seen in the airways after 24 h? A “yes” to all questions indicated abnormal clearance, while “no” to all indicated normal clearance. A regional impaired clearance was the case, if there were one or a few focal retentions at 24 h, while the other two parameters were normal. In other cases of inconsistencies, the test was read as inconclusive (see below).

**Fig. 2:**
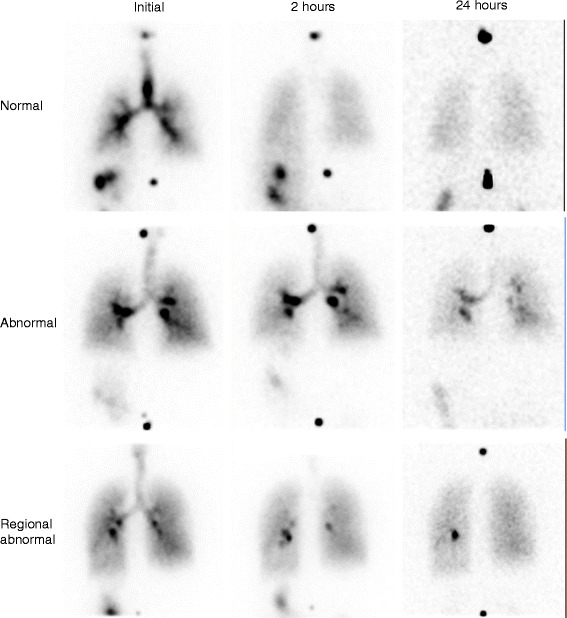
Three examples of posterior static acquisitions from a normal, an abnormal, and a regional abnormal PRMC test showing initial aerosol deposition together with remaining aerosol deposition after 2 and 24 h. Two ^57^Co markers over the cervical and lumbar spine are used to help position the patient. *Top row:* normal PRMC test. LR_2_ was 38 % (predicted = 70 %, upper normal limit = 86 %, *Z*-score = -3.3). *Centre row:* abnormal PRMC test. The radioaerosol is seen to be initially centrally deposited, and after 2 h, very little is cleared. The same foci are still very distinct after 24 h. LR_2_ was 100 % (predicted = 72 %, upper normal limit = 88 %, *Z*-score = 2.9). *Bottom row:* regional abnormal PRMC test. LR_2_ was 85 % (predicted = 77 %, Upper normal limit = 93 %, *Z*-score = 0.8), but a distinct focus is seen in the left lung after both 2 and 24 h

Generally, the interpretation of the PRMC tests can be divided into three main groupings: (1) where the test is clearly abnormal rendering PCD or SCD possible explanations, (2) where the test is inconclusive whereby PCD cannot be ruled out, or (3) where the test is normal or otherwise not consistent with PCD. If for example one or a few focal retentions were seen at 24 h, this would imply overall normal clearance with only regional impairment. Such a test would therefore be placed in group (3). As another example, some patients show signs of SCD but not PCD (e.g., slow clearance indicated by abnormal LR1 and/or LR2 but normal retention at 24 h and normal bolus transport in central airways), and such a test would as well be placed in group (3). This means that PRMC has the ability to discern between PCD and SCD in some but not all cases. Examples of static acquisitions from an abnormal, a normal, and a regional abnormal test, respectively, are seen in Fig. 2.

An inconclusive test is usually a result of one of the following causes: (1) cough during examination, (2) inconsistency between LR_1_ and LR_2_ values, bolus transport, and 24-h retention, or (3) very peripheral radioaerosol deposition, which can cause the predicted normal range for lung retention (pLR_1_ or pLR_2_ + 1.645 SD, respectively) to include a retention of 100 %. This problem is minimised by means of the inhalation technique described earlier, which serves to promote deposition of the radioaerosol in the central airways.

As noted, if the patient coughs during the first 2 h of the PRMC test, an apparently normal test will be interpreted as inconclusive. This is due to the fact that the clearance measured may be the result of cough clearance instead of clearance due to ciliary movement. Hence, an effective cough clearance would be able to disguise a true abnormal clearance leading the test to appear as being normal (i.e., false negative). In contrast, an abnormal test is still considered reliable despite coughing (i.e., true positive). Consequently, a staff member monitored the patients during the first 2 h of the test and registered time and number of coughs and throat clearing. Subsequently, all patients who had coughed in this timeframe had to perform a 1-min voluntary cough clearance test immediately after the 2-h acquisition, which served as a measure for the effectiveness of their coughing with regard to airway clearing. If this test showed minimal cough clearance, an otherwise normal PRMC test could be regarded as being normal provided that the cough during the first 2 h of the test had been very limited (say 1–2 small coughs or huffs). The voluntary cough clearance test is not thought to affect the interpretation of the 24-h scan, since this scan is predominantly determined by retention in the smaller bronchi, bronchioles, and possibly bronchiectasis which is only marginally affected by cough clearance, since coughing is thought mainly to affect the first six airway generations [17]. In addition, no restrictions are given to the patients with regard to coughing in the timeframe before the 24-h scan.

### Final clinical diagnosis

The final clinical diagnoses were primarily obtained via national electronic hospital records, in which we were able to search for each patient by means of their unique social security number. In a few cases, this information was complemented with non-electronic records from the National PCD Centre in Copenhagen. Results from EM and ciliary function testing were found in a database in the National PCD Centre in Copenhagen where these tests are being performed. A description of these two methods can be found in the ERS recommendations for PCD [18]. All patients in Denmark with a verified, definitive PCD diagnosis are followed on a regular basis in the National PCD Centre in Copenhagen. Therefore, these patients were easy to identify.

Optimally, all patients in the study should have had their tentative PCD diagnosis confirmed or refuted after the completion of both thorough clinical assessment, nasal NO measurement, EM, ciliary function testing, PRMC, and in some cases gene testing. Because of the set-up of the study, the different tests were done only when considered pertinent by the treating doctor, meaning that only some of the patients were examined this comprehensively.

For a minority of the patients (32 out of 239), a conclusion could not be reached with regard to their final clinical diagnosis. This was most often due to lack of information in the national electronic hospital records or due to on-going work up of a patient with contradicting test results.

### Statistical analysis

Two-sample *t* test for means was used for comparisons unless both groups were larger than 30, in which cases a *z* test was performed instead. Comparisons were done between PI in patients with a normal and abnormal PRMC test and between PI and *Z*-scores for LR_1_ and LR_2_ for children vs. adults with a normal PRMC.

### Ethics

The Danish Data Protection Agency has approved the study.

## Results

### Overview

In total, 239 patients with an age range of 4 to 75 years were included in this study (Table 1). 23.8 % had a positive PRMC test (PCD/SCD), 10.5 % had an inconclusive test while the remaining 65.7 % had a test that ruled out PCD. The PRMC results were rather uniformly distributed with regard to age with only the group of 55- to 75-year-old patients having a lower percentage of abnormal (7.1 %) and inconclusive results (3.6 %) than the other groups (abnormal ~20–35 % and inconclusive ~6–13 %). Consequently, they also had a higher percentage of results that were normal or otherwise not consistent with PCD.

**Table 1:** Distribution of PRMC results in relation to age

Results from PRMC test	Age group					
	4–14 years	15–24 years	25–34 years	35–54 years	55–75 years	All
	*n* = 105 (%)	*n* = 28 (%)	*n* = 34 (%)	*n* = 44 (%)	*n* = 28 (%)	*n* = 239 (%)
PCD/SCD	21 (20)	9 (32.1)	12 (35.3)	13 (29.5)	2 (7.1)	57 (23.8)
	[14, 67 %]	[3, 33 %]	[3, 25 %]	[3, 23 %]	[0]	[23, 40 %]
Inconclusive	14 (13.3)	3 (10.7)	2 (5.9)	5 (11.4)	1 (3.6)	25 (10.5)
	[4, 29 %]	[0]	[0]	[0]	[0]	[4, 16 %]
Normal or otherwise not consistent with PCD	70 (66.7)	16 (57.1)	20 (58.8)	26 (59.1)	25 (89.3)	157 (65.7)
	[0]	[0]	[0]	[0]	[0]	[0]

Numbers in square brackets show the number and percentage of patients in each group ending up with verified PCD as final clinical diagnosis

### PRMC results in relation to final clinical diagnosis

No patient with a PRMC test that ruled out PCD ended up with PCD as final clinical diagnosis (Table 2)—that is, there were no false negatives. However, 29 % of patients in this group ended up with an uncertain final clinical diagnosis.

**Table 2:** PRMC results in relation to final clinical diagnosis

	Final clinical diagnosis					
Results from PRMC test	Verified PCD	Uncertain but probably PCD	Uncertain	Uncertain but probably not PCD	Not PCD	Total
	*n* = 27 (%)	*n* = 3 (%)	*n* = 32 (%)	*n* = 28 (%)	*n* = 149 (%)	*n* = 239 (%)
PCD/SCD	23 (85.2)	2 (66.6)	7 (21.9)	3 (10.7)	22 (14.8)	57 (23.8)
Inconclusive	4 (14.8)	1 (33.3)	3 (9.4)	1 (3.6)	16 (10.7)	25 (10.5)
Normal or otherwise not consistent with PCD	0 (0)	0 (0)	22 (68.7)	24 (85.7)	111 (74.5)	157 (65.7)

The final clinical diagnoses of patients with an abnormal PRMC test were somewhat more variable. 40 % ended up with verified PCD while 39 % had the PCD diagnosis rejected—that is, 39 % of the positive PRMC tests were false positives. At the end of the study, 21 % had an uncertain diagnosis. A similar variability in the final diagnoses was seen amongst patients with an inconclusive PRMC test. PCD was rejected in 64 % of cases while it was verified in 16 %. At the end of the study, 20 % had an uncertain diagnosis.

The highest percentage of patients ending up with verified PCD as final clinical diagnosis were seen amongst children 4–14 years old (67 % of patients having an abnormal PRMC test) while no patients ≥55 years ended up with the final clinical diagnosis of PCD (Table 1). All four patients with an inconclusive PRMC test that ended up with PCD as final clinical diagnosis were ≤14 years. The reason for their PRMC tests being inconclusive was pronounced coughing resulting in LR_1_ and LR_2_ values within the normal range while bolus transport and 24-h static acquisition were abnormal.

### PRMC results in relation to ciliary function testing, EM, and final clinical diagnosis

As mentioned earlier, not all patients underwent the full work up for PCD whereby it has only been possible to compare PRMC results with ciliary function testing and EM in 108 and 79 cases, respectively (Tables 3 and 4). Seventy-two patients had both tests performed. As a whole, there was good concordance between the three different test results and the final clinical diagnoses. However, in some cases, significant discrepancies were seen between results. This was mainly (1) patients with a PRMC test ruling out PCD but with an abnormal EM or ciliary function test who ended up with a final clinical diagnosis rejecting PCD, (2) patients with an inconclusive PRMC test, an abnormal EM or ciliary function test, and a final clinical diagnosis rejecting PCD, (3) patients with an abnormal PRMC test but a normal EM who ended up with a final clinical diagnosis confirming PCD, or (4) patients with an abnormal PRMC test and abnormal EM or ciliary function test but with a final clinical diagnosis rejecting PCD. All cases with discrepant results have been marked with asterisk in Tables 3 and 4, and moreover, further information about each of these patients is presented in Table 5 (please note that some of the 23 marked patients in Tables 3 and 4 are the same so that they represent only 21 different patients).

**Table 3:** PRMC results in relation to ciliary function testing and final clinical diagnosis

	Results from ciliary motility study			Final clinical diagnosis related to results from PRMC and ciliary motility study				
Results from PRMC test	Abnormal, consistent with PCD	Inconclusive	Normal, not consistent with PCD	Verified PCD	Uncertain but probably PCD	Uncertain	Uncertain but probably not PCD	Not PCD
	*n* = 45 (%)	*n* = 17 (%)	*n* = 46 (%)	*n* = 25 (%)	*n* = 2 (%)	*n* = 7 (%)	*n* = 3 (%)	*n* = 71 (%)
PCD/SCD	26 (57.8)			22 (88)	1 (50)	–	–	3^a^ (4.2)
		2 (11.8)		–	–	–	–	2 (2.8)
			15 (32.6)	–	–	3 (42.8)	1 (33.3)	11 (15.5)
Inconclusive	8 (17.8)			3 (12)	–	1 (14.3)	–	4^a^ (5.6)
		2 (11.8)		–	1 (50)	–	–	1 (1.4)
			8 (17.4)	–	–	1 (14.3)	–	7 (9.9)
Normal or otherwise not consistent with PCD	11 (24.4)			–	–	1 (14.3)	–	10^a^ (14.1)
		13 (76.4)		–	–	1 (14.3)	2 (66.6)	10 (14.1)
			23 (50)	–	–	–	–	23 (32.4)

^a^Cases with discrepant results. Further information about each of these patients is presented in Table 5

**Table 4:** PRMC results in relation to EM and final clinical diagnosis

	Results from EM study			Final clinical diagnosis related to results from PRMC and EM study				
Results from PRMC test	Abnormal, consistent with PCD	Inconclusive	Normal, not consistent with PCD	Verified PCD	Uncertain but probably PCD	Uncertain	Uncertain but probably not PCD	Not PCD
	*n* = 23 (%)	*n* = 13 (%)	*n* = 44 (%)	*n* = 26 (%)	*n* = 2 (%)	*n* = 4 (%)	*n* = 2 (%)	*n* = 46 (%)
PCD/SCD	18 (78.3)			17 (65.4)	–	–	–	1 (2.2)
		5 (38.5)		2 (7.7)	–	1 (25)	–	2 (4.3)
			16 (36.4)	3^a^ (11.5)	1 (50)	1 (25)	–	11 (23.9)
Inconclusive	3 (13.0)			2 (7.7)	–	–	–	1^a^ (2.2)
		3 (23.0)		1 (3.8)	–	–	–	2 (4.3)
			7 (15.9)	1^a^ (3.8)	1 (50)	1 (25)	1 (50)	3 (6.5)
Normal or otherwise not consistent with PCD	2 (8.7)			–	–	1 (25)	–	1^a^ (2.2)
		5 (38.5)		–	–	–	1 (50)	4 (8.7)
			21 (47.7)	–	–	–	–	21 (45.7)

^a^Cases with discrepant results. Further information about each of these patients is presented in Table 5

**Table 5:** Elaboration of patients with significant discrepancies between test results

Variables	Patient					
	1	2	3	4	5	
Age, years	15	6	15	17	10	
PRMC	PCD/SCD	PCD/SCD (A normal PRMC was found 2 years later)	PCD/SCD	Inconclusive	Inconclusive	
Ciliary motility study	Abnormal (but some sequences are described as being normal)	Abnormal	Abnormal (described as only slightly abnormal and partly inconclusive test)	Abnormal (described as being only slightly abnormal)	Abnormal (described as being only slightly abnormal)	
EM	Normal	Abnormal	Normal	Normal	Abnormal (described as being only slightly abnormal)	
Nasal NO	Normal	Normal	Normal	–	Normal	
Final clinical diagnosis	Not PCD	Not PCD	Not PCD	Not PCD	Not PCD	
Comments on final clinical diagnosis	Recurring infections apparently due to immune deficiency	Today, asymptomatic	Unknown restrictive lung disease and recurring upper airway infections	Abnormal ciliary study might be due to SCD	Today, asymptomatic	
		Abnormal PRMC, ciliary study and EM might be due to SCD			Abnormal ciliary study and EM might be due to SCD	
	Abnormal PRMC and ciliary study might be due to SCD		Abnormal PRMC and ciliary study might be due to SCD			
Variables	Patient					
	6	7	8	9	10	
Age, years	20	34	61	8	13	
PRMC	Inconclusive	Inconclusive	Normal	Normal	Normal	
Ciliary motility study	Abnormal (described as being only slightly abnormal)	Abnormal (described as being only slightly abnormal)	Abnormal (described as being only slightly abnormal)	Abnormal	Abnormal (described as being only slightly abnormal)	
EM	–	Normal	–	Normal	–	
Nasal NO	–	Normal	–	Normal	–	
Final clinical diagnosis	Not PCD	Not PCD	Not PCD	Not PCD	Not PCD	
Comments on final clinical diagnosis	Recurring aspergilloma	Abnormal ciliary study might be due to SCD	Asymptomatic today	Asthma	Asthma	
				Today asymptomatic	Today, only few symptoms	
	Abnormal ciliary study might be due to SCD		Abnormal ciliary study might be due to SCD	Abnormal ciliary study might be due to SCD	Abnormal ciliary study might be due to SCD	
Variables	Patient					
	11	12	13	14	15	
Age, years	10	11	7	8	13	
PRMC	Normal	Normal	Normal	Normal	Normal	
Ciliary motility study	Abnormal (the specimen was infected)	Abnormal	Abnormal (described as being only slightly abnormal)	Abnormal	Abnormal	
EM	Normal	Abnormal	–	Normal	Normal	
Nasal NO	Normal	Normal	Normal	Normal	Normal	
Final clinical diagnosis	Not PCD	Not PCD	Not PCD	Not PCD	Not PCD	
Comments on final clinical diagnosis	Severe asthma and atopic dermatitis	Today, asymptomatic	Asthma and allergies	Today asymptomatic	Abnormal ciliary study might be due to SCD	
	Abnormal ciliary study might be due to SCD	No explanation has been found for the patient’s earlier airway symptoms	Today, only few airway symptoms	Abnormal ciliary study might be due to SCD		
		Abnormal ciliary study and EM might be due to SCD	Abnormal ciliary study might be due to SCD			
Variables	Patient					
	16	17	18	19	20	21
Age, years	24	5	7	9	37	12
PRMC	Normal	Normal	PCD/SCD	PCD/SCD	PCD/SCD	Inconclusive
Ciliary motility study	Abnormal	Abnormal (described as being only slightly abnormal)	Abnormal	Abnormal	Abnormal	Abnormal
EM	–	Normal	Normal (described as being partly inconclusive)	Normal	Normal (described as being partly inconclusive)	Normal
Nasal NO	Normal	Normal	–	–	–	Abnormal
Final clinical diagnosis	Not PCD	Not PCD	Verified PCD	Verified PCD	Verified PCD	Verified PCD
Comments on final clinical diagnosis	Abnormal ciliary study might be due to SCD	Asthma	Clinically PCD is plausible with frequent airway infections	Clinically PCD is plausible with frequent airway infections and severe basal bronchiectasis	Clinically PCD is plausible with frequent airway infections	Clinically PCD is plausible with chronic productive coughing, bronchiectasis and situs inversus
		Abnormal ciliary study might be due to SCD				

### Inconclusive tests

In our study, 90 % of patients had a conclusive result after just one PRMC test.

Unfortunately, only 10 out of 25 patients with an initially inconclusive PRMC test had a new PRMC test performed. However, 8 out of these 10 patients obtained a conclusive test after repeating the PRMC test once. Two patients needed more than one repetition.

### Comparison of penetration index and retention in different groups

When comparing mean PI from patients with a normal and abnormal PRMC test, respectively, we found no significant difference between the two groups suggesting that a more peripheral initial deposition was not the cause of the observed abnormal clearance (mean PI for normal PRMC = 0.478, mean PI for abnormal PRMC = 0.524, CI for difference in means = (-0.0189; 0.111)).

**Fig. 3:**
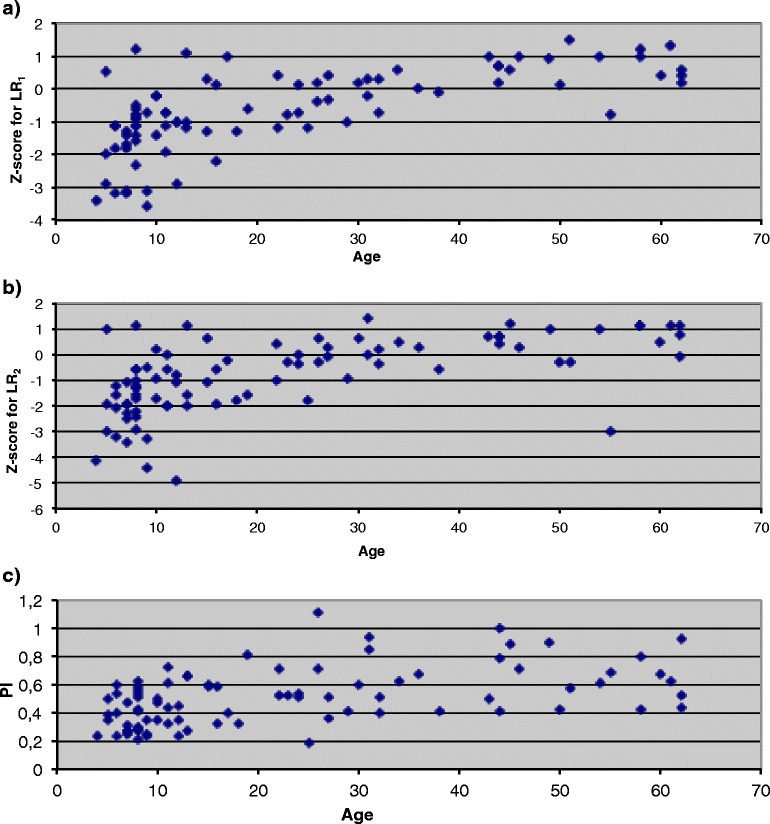
**a** Retention *Z*-score for LR_1_ in relation to age in patients with a perfectly normal PRMC test (this is defined as a normal PRMC test showing no signs of regional impaired clearance or slow clearance in any part of the lung in combination with no coughing or throat clearing during the 2-h PRMC test). Lower retention *Z*-scores represent better clearance. **b** Retention *Z*-score for LR_2_ in relation to age in patients with a perfectly normal PRMC test. Lower retention *Z*-scores represent better clearance. **c** PI in relation to age in patients with a perfectly normal PRMC test. Higher PI represents more peripheral initial deposition of the radioaerosol

The relation of retention *Z*-scores and PI to age was compared amongst patients who had a perfectly normal PRMC test (Fig. 3a–c). When comparing children (5–14 years) with adults (>14 years), significant differences were found in the case of retention *Z*-scores for LR_1_, retention *Z*-scores for LR_2,_ and PI (Table 6).

**Table 6:** Comparison of retention *Z*-scores and PI between children (5–14 years) and adults (>14 years)

	Mean values for children	Mean values for adults	Difference of the means (95 % CI)	*p* value
	*n* = 42	*n* = 44		
*Z*-score for LR_1_	−1.4	0.089	1.47 (1.05; 1.89)	*p* < 0.001
*Z*-score for LR_2_	−1.7	−0.0023	1.65 (1.15; 2.15)	*p* < 0.001
PI	0.40	0.61	0.2 (0.127; 0.276)	*p* < 0.001

All the included patients had a perfectly normal PRMC test. (This is defined as a normal PRMC test showing no signs of regional impaired clearance or slow clearance in any part of the lung in combination with no coughing or throat clearing during the 2-h PRMC test)

## Discussion

With its 239 patients, this is by far the largest study to date concerning clinical use of the PRMC test. Furthermore, it is the first study presenting a large data set from PRMC tests used as part of everyday clinical work up of patients suspected of having PCD.

According to our study, the main strength of the PRMC test is its ability to rule out an otherwise suspected PCD diagnosis since the study showed that no patient with a normal PRMC test or a PRMC test otherwise not consistent with PCD ended up having PCD as final clinical diagnosis—that is, we experienced no false negatives. Only 10 % of tests were inconclusive, and of these, only 16 % had a final clinical diagnosis of PCD. The fact that no patients >14 years with an inconclusive test result ended up with a definite PCD diagnosis means that even an inconclusive test in adults strongly suggests that PCD is very unlikely.

To this date, there are only few studies on the usage of PRMC in relation to PCD. Most of these studies deal only with a very limited number of patients and especially the number of PCD patients in each study is sparse [19–22]. However, these studies do show markedly reduced tracheobronchial clearance in patients with known PCD compared with healthy individuals which is in alignment with our results. Most recently, this was found by Walker et al. in a comparison of six PCD patients and four healthy controls [23]. In our earlier large study [14], 95 patients underwent PRMC. Fifty-seven of these patients had known PCD or were diagnosed during the course of the study. The study was divided into three sequentially performed substudies: (1) a cross-sectional study of patients with known PCD which showed abnormal PRMC in 14 out of 15 patients (one PRMC was inconclusive), (2) a prospective blinded trial in which PRMC was tested against EM and ciliary function testing which showed good concordance between the different methods, and (3) an implementation study using PRMC as part of the routine work up of 21 patients referred for PCD investigation. It was concluded that in a selected group of patients, PRMC would be a good supplementary test in the diagnosis and exclusion of PCD. These observations were generally corroborated by our present results, which were obtained from a very large group of patients in which the PCD diagnosis was not established beforehand. Even though the described implementation study with 21 patients should assess PRMC as a routine method in PCD work up, one might think that the selection of patients for the trial have been affected by the fact that this new method was suddenly available to a large group of patient suspected of having PCD. One might speculate that patients with the highest suspicion of PCD would then be referred to the PRMC test first. This is in line with the fact that 6 out of 21 (29 %) had PCD as final clinical diagnosis compared with 11 % in the present study. Therefore, we think that our present study better represents what to expect from the PRMC test if implemented in the everyday work up of suspected PCD patients. In addition, because of the large number of patients included in this study, for the first time, we have been able to analyse data from 42 children presenting perfectly normal PRMC tests in order to assess the reference material used today.

### PRMC results in relation to final clinical diagnosis

As mentioned, one of the biggest strengths of the PRMC test is its ability to rule out PCD. This is supported by the fact that we found no false negatives in the present study.

In addition, in a selected group of patients apparently the test has the ability to point out patients that are likely to have PCD. In our study population, when the PRMC test was abnormal, 40 % ended up having PCD as final clinical diagnosis. This means that an abnormal test to some degree supports the PCD diagnosis, though it is important to remember that the ability to diagnose PCD is not the main advantage of the test.

Another reason, why the PRMC test is a useful clinical tool, is its high degree of conclusive results. After just one test, 90 % of patients had a conclusive result (Table 1). Eighty percent of patients initially having an inconclusive result obtained a conclusive test after only one repetition. If looking at the subgroups of patients on which either ciliary function testing or EM was performed, the rates of conclusive results after the first test were as follows: 69 % of ciliary function tests were conclusive compared with 83 % of PRMC tests. Seventy-five percent of EM tests were conclusive compared with 84 % of PRMC tests.

As seen in Table 1, by far the largest number of patients having PCD as final clinical diagnosis was found in the group of patients being 4–14 years old and having an abnormal PRMC test while none over the age of 55 was diagnosed with PCD. This is in line with the clinical presentation of the disease starting in early childhood and most often resulting in severe airway symptoms often leading to early work up.

Generally, the results from ciliary function testing and EM were in good concordance with results from the PRMC tests and the final clinical diagnoses. However, some discrepancies were seen. Each of these cases has been elaborated further, and they are all presented in Table 5. In 17 out of the 21 cases (patient No. 1–17), the observed discrepancy is thought to be due to SCD causing false positive tests. In 10 out of these 17 cases (patient No. 8–17), the patient had a PRMC test not consistent with PCD while the ciliary function testing was abnormal. The fact that SCD could affect the results of ciliary function testing and not PRMC could be explained in two ways: First, the different tests are not necessarily performed at the same point in time, which means that the degree of SCD affecting the patient might differ between the two tests. Second, and more importantly, one of the great advantages of the PRMC test is that it is not affected by nasal SCD because the PRMC test examines the ciliary function of the entire lung and not just the nasal ciliary function.

In four other cases (patient No. 18–21), a normal EM was found while the rest of the tests were abnormal together with a final clinical diagnosis confirming PCD. This is in line with a growing number of publications showing normal EM tests in PCD patients [2, 9, 24, 25, 26].

### Penetration index and *Z*-scores

We found no significant difference between the initial aerosol distribution in the lungs (indicated by the mean PI) in patients with a normal and abnormal PRMC test. The magnitude of PI is accounted for when calculating predicted values for LR_1_ and LR_2_. But if PI had been significantly higher in patients with an abnormal PRMC test, this would have implied that we did not to a satisfactory degree take into account the magnitude of PI when calculating predicted values. Fortunately this does not seem to be the case, which means that abnormal test results are most likely caused solely by slow clearance of the radioaerosol.

When comparing children and adults presenting perfectly normal PRMC tests, our results show markedly faster clearance (i.e., lower *Z*-scores for LR_1_ and LR_2_) in children than in adults. This might be an expression of the fact that children under 14 years of age have smaller lungs and thereby generally shorter airways than adults [27]. Assuming that the cilia in healthy children and adults work equally effective, it makes sense that shorter airways means faster overall lung clearance.

It is also noteworthy that the mean PI in these children is significantly lower (i.e., more central initial lung deposition) than the mean PI seen in the corresponding adults. According to the discussion above, this should not affect the results of the test seeing that the magnitude of PI is accounted for in the calculation of predicted LR_1_ and LR_2_. Nevertheless, it raises the question if the formulas for calculating predicted LR_1_ and LR_2_ to a satisfactory degree take the magnitude of PI into account when used on children under 14 years of age. If not, at least to some degree, the low PI seen amongst the children might explain the faster clearance observed. If the fast clearance observed in children is primarily due to smaller lungs, it would make sense to incorporate, e.g., the height of the patient when calculating PI. As of today, PI only takes into account the *proportion* of the lung receiving a certain degree of radioaerosol. Thereby, the actual size of the lungs and thus the length of the airways are not accounted for.

Whatever the explanation, according to our results, it seems fair to state that the reference material used for the PRMC test today does not in a satisfactory degree take into account the effect of age (or height) on lung clearance, which is most evident for children under 14 years of age. This is not surprising since our reference material is based upon a cross-sectional study of 53 healthy never-smoking adults (18 to 84 years) meaning that predictive norms have been extrapolated for ages <18. In the reference material, the dependency of age on PRMC was weak [16]. But according to the present study, this could be different if one incorporates children <14 years in such a reference material. To date, no reference material on PRMC in children exists.

### Algorithm for work up of PCD in specialised centres with access to PRMC testing

**Fig. 4:**
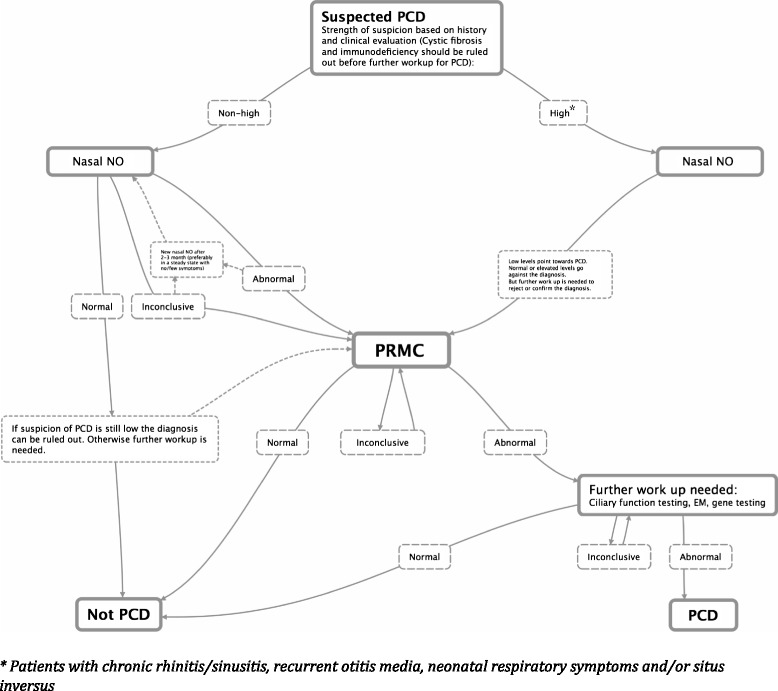
Suggested algorithm for the work up of patients suspected of having PCD. The algorithm presupposes work up in a specialised centre with access to PRMC testing. In our patient population, the PRMC test would obviate the need for further work up in relation to PCD in 2/3 of the patients

Based on our experience with work up of patients suspected of having PCD, we propose the following algorithm for PCD investigation (Fig. 4).

When patients have been referred to a specialist centre with expertise in PCD work up, the ensuing work up should take into account the strength of suspicion of PCD based on history and clinical presentation of the patient [28]. However, whether the initial suspicion is high or non-high, measurement of nasal NO levels should be used as a first wave work up tool. The test is easy to perform and non-invasive and is suitable as a targeted case finding tool for PCD [2]. In patients with a normal or high level of nasal NO together with a non-high suspicion of PCD, the diagnosis could be ruled out. If however the suspicion is high, further work up for PCD should be performed since a few studies have shown normal or elevated levels of nasal NO in patients with known PCD [11, 13, 29]. A low level of nasal NO points towards PCD, but other conditions such as cystic fibrosis [30], panbronchiolitis [31], nasal polyposis [32], and chronic sinusitis [32] have also been shown to demonstrate reduced levels of nasal NO. Therefore, a measurement of low nasal NO should always be followed by further work up to confirm or reject the diagnosis. This work up should amongst other things include a new nasal NO measurement after, e.g., 2–3 months—preferably in a steady state with no or few symptoms apart from those to be expected from the disease. If rising or normal values can then be attained, the diagnosis might be rejected [33, 34].

Patients with initially high suspicion of PCD and all patients with abnormal or inconclusive nasal NO values should have PRMC performed. If the PRMC test is normal, PCD can be ruled out in accordance with the fact that we found no false negatives in the present study. In our setting, PCD could be ruled out in roughly two out of three patients. An inconclusive test would require a re-test (in the present study 80 % acquired a conclusive result after only one re-test) while an abnormal test should lead to more intensive work up with EM, ciliary function testing, and possibly gene testing.

### Strengths and weaknesses of the study

One of the main strengths of the present study is that it shows what to expect of the PRMC test if implemented in the everyday clinical work up of PCD. Another strength is the large number of patients that are included. By far, it is the largest study to date concerning clinical use of the PRMC test. Furthermore, even when taking into account the weaknesses mentioned in the following, the fact that no patient with a normal PRMC test ended up with PCD as final clinical diagnosis is quite convincing. It is also worth noticing the very high rate of conclusive results achieved with the PRMC test.

The main weakness of this study is that all of the PRMC results have been used in the subsequent work up of the patients, which means that these results have inevitably affected the final clinical diagnoses. In addition, not all patients underwent the same thorough work up for PCD. This was due to the fact that in some patients, the assumed PCD diagnosis was easily rejected leaving some tests unnecessary in the everyday clinical work up for PCD. It would of cause be advantageous to make a similar study with blinded results and with strict protocols for the work up of all patients suspected of having PCD. On a smaller scale, this was done by Marthin et al. [14] in our substudy 2, which included 59 patients, and as mentioned, those results are generally in line with the results presented in the present work. Another weakness of the present study is the lack of systematically collected clinical information concerning smoking habits, airway symptoms, and pulmonary diseases that might influence mucociliary clearance. On the other hand, we know that all patients in general had unexplained airway symptoms (or else they would not have been referred to the PRMC test) and that they as far as possible had been without acute airway infection 4–6 weeks prior to the test as already explained.

## Conclusions

In this study, we have evaluated the use of the PRMC test in the work up of patients suspected of having PCD. To this date, it is the only method for testing the mucociliary function of the lower airways, and when compared to other existing tests, one of its great strengths is that it is not affected by secondary mucociliary defects affecting nasal cilia. In addition, the test is non-invasive and is applicable to children as young as 5 years of age. The method is relatively easy to introduce and perform and could therefore potentially be of interest for general nuclear medicine departments if used for research purposes such as to investigate possible links between mucociliary dysfunction and pathophysiology of lung diseases or pharmacological challenges to the mucociliary apparatus [1]. But if used in the work up of patients suspected of having PCD, the interpretation of tests needs expertise in addition to a relatively large population of patients (in concordance with the infrequent nature of the disease). However, it is yet to be established if these tests/results can be replicated in less-experienced nuclear medicine facilities after a proper amount of training.

From the above, we can conclude the following: (1) in the present study, we found no false negatives. We therefore believe that PRMC has the ability to reject a suspected PCD diagnosis with great certainty, when used in daily clinical work up (in the present study in 2/3 of referred patients). (2) PRMC has the ability to find candidates for further work up. In the present study, 40 % of patients with an abnormal PRMC test ended up having PCD as final clinical diagnosis. This number will of course depend greatly upon the group of patients being investigated. (3) PRMC has a high rate of conclusive results (90 % in the present study). (4) In addition, our results indicate that there might be a need for reference material for children.
